# Achieving consensus over the assessment of clinical signs in eczema trials

**DOI:** 10.1186/1745-6215-16-S1-P19

**Published:** 2015-05-29

**Authors:** Kim S Thomas, Jochen Schmitt, Phyllis I Spuls, Eric Simpson, Masutaka Furue, Stefanie Deckert, Christian Apfelbacher, Joanne Chalmers, Hywel C Williams

**Affiliations:** 1 Centre of Evidence Based Dermatology, University of Nottingham, Nottingham NG9 2NR, UK; 2 Centre for Evidence-based healthcare, Medical Faculty Carl Gustav Carus, TU Dresden, Germany; 3 Department of Dermatology, Academic Medical Centre, University of Amsterdam, Amsterdam, The Netherlands; 4 Department of Dermatology, Oregon Health & Sciences University, Portland, Oregon, U.S.A; 5 Department of Dermatology, Kyushu University, Fukuoka, Japan; 6 Medical Sociology, Institute of Epidemiology and Preventive Medicine, University of Regensburg, Germany

## Background

Eczema (syn. atopic dermatitis, atopic eczema) is a chronic, itchy, skin disease that commonly starts in childhood. Over 500 randomised controlled trials of eczema treatment have been conducted, but a core outcome set is lacking, leading to inefficiency and waste.

The Harmonizing Outcome Measures for Eczema (HOME) initiative seeks to agree a core set of outcomes for future research, and has previously agreed key outcome domains: clinical signs, patient-reported symptoms, quality of life and long-term control.

## Materials and methods

Two systematic reviews were conducted as per the HOME methodology: i) review of outcome measures used to capture clinical signs, and ii) review of validation studies of the identified scales. Results of these reviews and other data were presented at a face-to-face consensus meeting.

The principle focus of the HOME III meeting, (April 2013, San Diego, USA), was to achieve consensus over the measurement of eczema clinical signs. The meeting included 56 participants (clinicians, patients, researchers and industry representatives) from 10 countries including Asia, Europe, South America, and the US. Consensus methodologies included: presentation of evidence, small and large group discussion and anonymous key-pad voting with pre-defined consensus criteria.

## Results

Of the 16 identified scales that assess eczema clinical signs, the Eczema Area and Severity Index (EASI) and the Scoring Atopic Dermatitis index (SCORAD) were identified as valid and reliable. The EASI has adequate validity, responsiveness, internal consistency, and intra-observer reliability. The SCORAD has adequate validity, responsiveness, inter-observer reliability, but unclear intra-observer reliability. HOME III delegates agreed that EASI was the preferred instrument to measure the core outcome of eczema signs in future AE-trials (90% of delegates in favour of EASI).

## Conclusions

Those involved in designing, reporting and using evidence from clinical trials on eczema are asked to comply with this consensus to enable better evidence-based decision making and improved patient care.

